# Supplementary material for: Genome-Wide Association Study Identified a Quantitative Trait Locus and Two Candidate Genes on Sus scrofa Chromosome 2 Affecting Vulvar Traits of Suhuai Pigs
Source: Genes (Basel). 2022 Jul 22;13(8):1294. doi: 10.3390/genes13081294 (PMC9330916; doi:10.3390/genes13081294)
Supplement: Supplementary file 1 [file genes-13-01294-s001.zip › genes-1793781-supplementary.pdf]

# Genome-wide association study identified a quantitative trait locus and two candidate genes on *Sus scrofa* chromosome 2 affecting vulvar traits of Suhuai pigs

**Yanzhen Yin [1,2], Liming Hou [1,2,3] Chenxi Liu [1,2], Kaijun Li [1,2], Hao Guo [1,2], Peipei Niu [3], Qiang Li [4], Ruihua Huang [1,2,3], and Pinghua Li [1,2,3,*]**

Genes journal

Supplementary material:

Material S1: The code for quality control.

The code is as follows:

plink --file file –recode --geno 0.1 --mind 0.1 --maf 0.05 --allow-extra-chr --autosome --out out;

where --file refers to the genotypical data; --geno 0.1 refers to removing the single nucleotide polymorphism (SNP) with genotype frequency < 0.9; --mind 0.1 refers to removing the sample with call rate < 0.9; --maf 0.05 refers to the SNP with minor allele frequency < 0.05. --allow-extra-chr refers to the extra chromosome codes by name; --autosome refers to excluding all unplaced and non-autosomal SNPs.

Material S2: The code for imputation.

The code is as follows:

(1) plink --file target --recode vcf-iid --out target;

(2) java -Xmx120g -jar beagle.25Mar22.4f6.jar gt=target.vcf out=target;

(3) java -Xmx120g -jar beagle.25Mar22.4f6.jar gt=ref.vcf out=ref;

(4) java -Xmx120g -jar beagle.25Mar22.4f6.jar ref=ref.vcf.gz gt=target.vcf.gz out= impute;

(5) zcat impute.vcf.gz | java -Xmx120g -jar SnpSift.jar filter "(DR2>=0.9)" > Impute_0.9.vcf.gz;

(6) plink --vcf impute_0.9.vcf.gz --recode --maf 0.05 --out impute_0.9_0.05;

where the code is conducted in Linux; (1) the plink format file is converted to a vcf file; (2) genotype data of target population is phased; (3) genotype data of reference population is phased; (4) genotype data of target population is imputed using the reference population as the reference panel; (5) imputed loci with DR2 < 0.9 are filtered; (6) imputed loci with maf < 0.05 are filtered.

Material S3: The code for calling SNPs of re-sequenced data.

The code is as follows:

(1) fastp -i sample_1.fq.gz -I sample_2.fq.gz -o sample_1_clean.fq.gz -O sample_2_clean.fq.gz -c -q 20 -u 50 -n 15 -5 20 -3 20 -w 16 -h sample_clean.html -j sample_clean.json;

(2) bwa mem -t 100 -M -R "@RG\tID:sample\tLB:sample\tPL:illumina\tPU:sample\tSM:sample" "./Sus_scrofa11.1.fa" sample_1_clean.fq.gz sample_2_clean.fq.gz | samtools view -q 1 -Shb - > sample.bam;

(3) samtools sort sample.bam -@ 8 -m 8G -o sample_sorted.bam;

(4) samtools depth sample_sorted.bam | awk '{sum+=$3} END {print i "Average = " sum/NR}' i=sample_sorted >> depth.txt;

(5) java -Xmx240g -jar picard.jar MarkDuplicates MAX_FILE_HANDLES_FOR_READ_ENDS_MAP=8000 INPUT=sample_sorted.bam OUTPUT=sample_sorted.dedup.bam METRICS_FILE=sample_sorted.dedup.metrics VALIDATION_STRINGENCY=LENIENT;

(6) samtools index sample_sorted.dedup.bam;

(7) gatk --java-options "-Xmx240g" BaseRecalibrator -R "./Sus_scrofa11.1.fa" -I sample_sorted.dedup.bam --known-sites "./Sus_scrofa11.1.vcf" -O recal_data_sample.table;

(8) gatk --java-options "-Xmx240g" ApplyBQSR --bqsr-recal-file recal_data_sample.table -R "./Sus_scrofa11.1.fa" -I sample_sorted.dedup.bam -O sample_sorted.dedup.BQSR.bam;

(9) gatk --java-options "-Xmx240g" HaplotypeCaller -R "./Sus_scrofa11.1.fa" --native-pair-hmm-threads 100 -I sample_sorted.dedup.BQSR.bam -ERC GVCF -O sample_g.vcf;

(10) gatk --java-options "-Xmx240g" CombineGVCFs -R ./Sus_scrofa11.1.fa" --variant input.list -O combined.g.vcf;

(11) gatk GenotypeGVCFs -R "./Sus_scrofa11.1.fa" -V combined.g.vcf -G StandardAnnotation -O raw_varians.vcf;

(12) gatk SelectVariants -select-type SNP -V raw_varians.vcf -O raw_varians_snp.vcf;

(13) gatk VariantFiltration -V raw_varians_snp.vcf --filter-expression "QD <2.0 || MQ <40.0 || FS > 60.0 || SOR > 3.0 || MQRankSum < -12.5 || ReadPosRankSum < -8.0" --filter-name "PASS" -O raw_varians_snp.filter.vcf;

where the code is conducted in Linux; (1) raw data is filtered by fastp software; (2) filtered data is aligned with Sus scrofa 11.1 genome; (3) the result files of the previous step are sorted using samtools software; (4) sequencing depth is counted by samtools software; (5) duplicate reads are removed by picard software; (6) the result files of the previous step are indexed by samtools software; (7) the result files of the previous step are sorted again by gatk software; (8) base quality is recalibrated by gatk software; (9) gvcf files are generated in this step; (10) gvcf files of multiple samples are combined by gatk software; (11) all types of variants are extracted by gatk software; (12) SNPs are extracted by gatk software; (13) SNP files are filtered by gatk software.

Material S4: The code for GWAS.

The code is as follows:

(1) ldak --thin thin --bfile input --window-prune .98 --window-kb 100;

(2) awk < thin.in '{print $1,1}' > weights.thin;

(3) ldak --calc-kins-direct kinship --bfile input --weights weights.thin --power -.25;

(4) ldak --linear output --pheno phenotype –covar covar --bfile input --grm kinship;

where --thin refers to using the LDAK-Thin Model; --bfile represents the binary file formed by Chip data; --window-prune .98 represents the pruning threshold is 0.98; --window-kb 100 refers to the window size is 100 kb; --calc-kins-direct kinship refers to computing the kinship matrix using the direct method; --weights weights.thin refers to adding the weights file obtained in the previous step; and --power -.25 represents the power is -0.25; --linear refers to performing linear regression; output represents the result file; --pheno represents the phenotype file; --bfile represents the binary file formed by Chip or imputed data; kinship is the kinship file formed by the ldak program based on Chip or imputed data.

Material S5: The code for plots of Manhattan and Q-Q.

The code is as follows:

```
CMplot(gwasdata,plot.type=c("m","q"),LOG10=TRUE,ylim=NULL,threshold=c(1/nrow(gwasdata),0.05/nrow(gwasdata)),threshold.lt=c(1,2),threshold.lwd=c(1,1),threshold.col=c("black","grey"),amplify=TRUE,bin.size=1e6,chr.den.col=NULL,signal.col=c("red","green"),signal.cex=c(1,1),signal.pch=c(19,19),file="jpg",memo="",dpi=300,file.output=T,verbose=TRUE)
```

where the code is conducted by R software; the gwasdata file consists of four columns of data, and the headers of the four columns of data are SNP,

Chromosome, Position, and P in order.

Material S6: The code for plot of LD decay.

The code is as follows:

PopLDdecay -InVCF SNP.vcf -OutStat LDdecay -MaxDist 2000

where -InVCF represents the vcf file formed by Chip data; -MaxDist represents the maximum calculated distance; -OutStat represents the result file.

Material S7: The code for LDLA.

The code is as follows:

Step 1 constructs haplotypes based on genotype data and is conducted in Linux:

(1) plink --bfile sample --chr 2 --transpose --recode --out sample_tchr2;

cat sample_tchr2.tped | awk '{printf "M " $2;for(i=5;i<=NF;i+=1) {printf " " $i};printf "\n"}' > sample_chr2.bgl;

(2) java -jar beagle.jar unphased=sample_chr2.bgl missing=0 out=beagle scale=2.0 shift=0.1;

(3) gunzip beagle.sample_chr2.bgl.phased.gz;

(4) java -jar beagle.jar data=beagle.sample_chr2.bgl.phased scale=2.0 shift=0.1;

(5) java -jar pseudomarker.jar dag=beagle.sample_chr2.bgl.phased.dag.gz out=sample_states_chr2.txt;

(6) awk '{for(i=3;i<=NF;i++)printf"%s ", $i} {print ""}' sample_states_chr2.txt >

hap2_chr;

(7) awk '{print $2}' sample_states_chr2.txt > makersid_chr2;

(8) awk '{print $1 " " $2 " " $4}' sample.map > Mkidpos;

(9) awk '{print $2}' sample.fam > modified_id;

Step 2 performs LDLA analysis and is conducted by R software:

```
phe_name <- "vl"

chrs <- c(2)

LALD <- function(formula,phe,hap,fix) {

if (typeof(formula)=="language") {

class(formula) <- 'character'

formula <- paste(formula[2],formula[1],formula[3])}

numPar <- unlist(strsplit(formula,'~'))

hap <- as.data.frame(hap)

phe <- as.data.frame(phe)

fix <- as.data.frame(fix)

colnames(phe)[1] <-'id'

colnames(hap)[1] <- 'id'

ncolPhe <- ncol(phe)

ncolhap <- ncol(hap)

hap <- merge(hap,phe,by='id')

hap <- hap[order(hap$id),]

phe <-hap[seq(1,nrow(hap),2),ncolhap:ncol(hap)]
```

```
hap <- hap[,3:ncolhap]

attach(phe)

test_oneMK <- function(hapone){

if (max(hapone)==1)    {

res <- c(0,0,0,0,1)

} else {

Z <- matrix(0,nrow=nrow(phe),ncol=max(hapone))

Z[cbind(1:nrow(phe),hapone[seq(1,length(hapone),2)])] <-

Z[cbind(1:nrow(phe),hapone[seq(1,length(hapone),2)])] + 1

Z[cbind(1:nrow(phe),hapone[seq(2,length(hapone),2)])] <-

Z[cbind(1:nrow(phe),hapone[seq(2,length(hapone),2)])] + 1

rm.col <- which(colSums(Z)<1)

if (length(rm.col)>0){

Z <- Z[,-rm.col]}

if(length(numPar)>1){

H1=lm(as.formula(paste(formula,'+fix[,1]+fix[,2]+Z',sep='')),na.action='na.omit'

)

      } else {

      H1 <- lm(as.formula(paste(numPar[1],' ~ Z',sep='')),na.action='na.omit')}

res <-anova(H1)

res<c(res$F[rownames(res)=='Z'],res$Df[rownames(res)=='Z'],res$Df[rowname

s(res)=='Residuals'],res$Sum[rownames(res)=='Z']/sum(res$Sum),res$Pr[rown
```

```
ames(res)=='Z'])
res}}
myresult <- apply(hap,2,test_oneMK)
myresult <- t(myresult)
detach(phe)
colnames(myresult) <- c('Fvalue','Df1','Df2','var_pct','pval')
return(myresult)}
phefilename <- paste("phe-",phe_name,".txt",sep = "")
phe <- read.table(phefilename,header=T)
colnames(phe) <- c("id", "testphe")
fix <- read.table("fix.txt",header=F)
hapfilename <- paste("hap",chrs,"_chr",sep = "")
hap1 <- read.table(hapfilename)
hap2 <-t(hap1)
id1 <- read.table("modified_id")
id <- rep(id1[,1],each=2)
org<- rep(c(1,2),nrow(phe))
hap <- data.frame(id,org,hap2)
test <- LALD(formula=testphe~1,phe,hap,fix)
makersidfilename <- paste("makersid_chr",chrs,sep = "")
mkid <- read.table(makersidfilename)
test1 <- data.frame(mkid,test)
```

```
colnames(test1)[1] <- "id"

mkid.pos <- read.table("Mkidpos")

colnames(mkid.pos) <- c("chr","id","pos")

test2 <- merge(mkid.pos,test1,by="id")

test2filename <- paste(phe_name,"-chr",chrs,"-ldla-test2.txt",sep="")

write.table(test2,test2filename,row.names=F,quote=F)
```

Step 3 draws line chart of LDLA analysis and is conducted by R software:

```
library("ggplot2")

library("ggthemes")

alldat_k <- read.table("vlchr2.txt",header=T)

alldat_k$pos <- alldat_k$pos/1e6

alldat_k$pval <- -log10(alldat_k$pval)

pmin=max(alldat_k$pval)-2

min=3.48

max=3.97

ggplot(alldat_k,aes(pos,pval))+

geom_line(size=1,linetype =1,color="#56B4E9")+

labs(y=expression(-Log[10]*P), x="Position (Mb)", title="LDLA")+

theme_par()+theme(plot.title = element_text(hjust = 0.5),axis.title.x =
element_text(size = 20),axis.title.y = element_text(size = 20))+

geom_vline(xintercept=c(min,max),color="red",size=0.8,linetype=3)+geom_hli
ne(yintercept=pmin,color="red",size=0.8,linetype=3)
```

```
ggsave("vlchr2.png",width = 12,height = 8,limitsize=F)
```

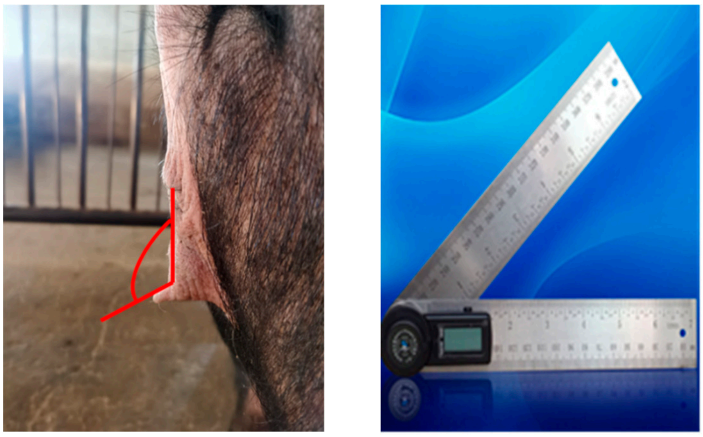

Figure S1. The real pictures of vulvar traits. The left plot was the real picture of VA of Suhuai pigs and the right plot was protractor.

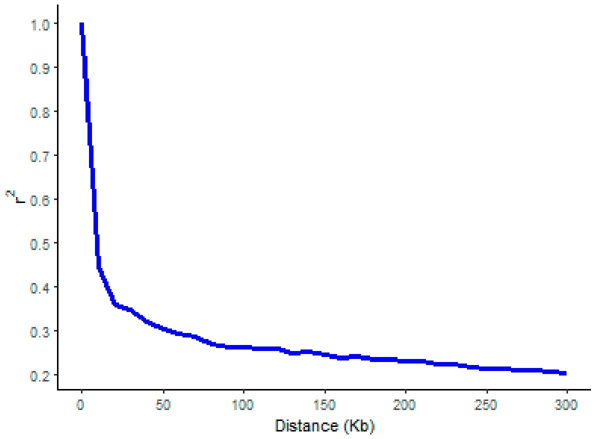


Figure S2. LD decay plot of 270 Suhuai pigs by Chip data. Calculate the r2 mean value for a window per 10 Kb.

(a) (b)

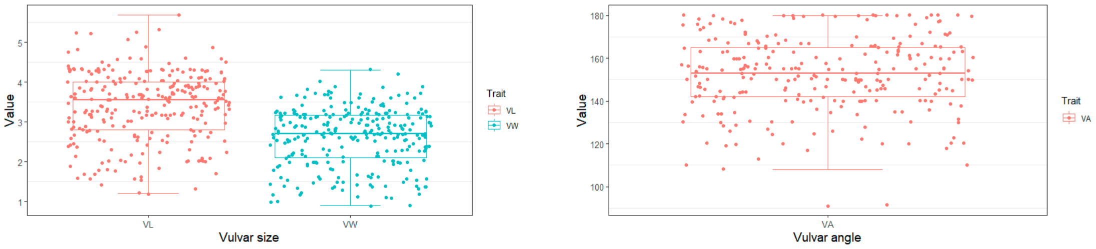


Figure S3. Box plot of vulvar phenotypes of Suhuai pigs. (a) Data distribution

of VL and VW of 270 Suhuai pigs. (b) Data distribution of VA of 258 Suhuai pigs.

(a) (b)

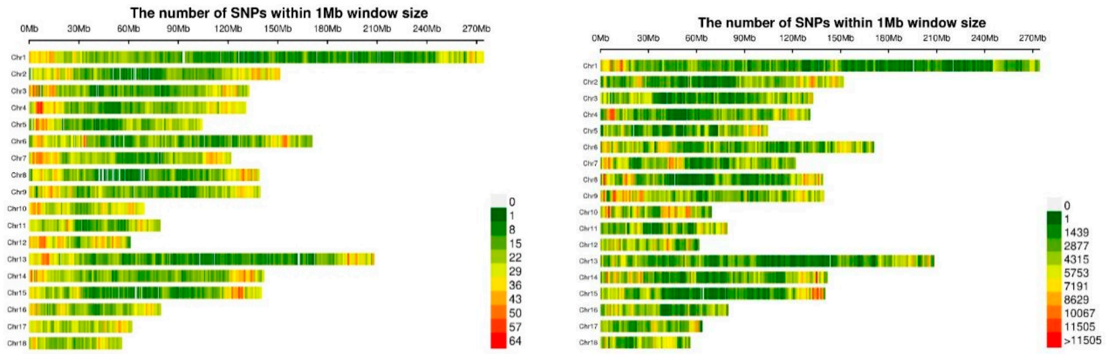


Figure S4. Density map of SNPs. (a) The data was collected from Chip data. (b) The data was collected from imputed data.

(a)

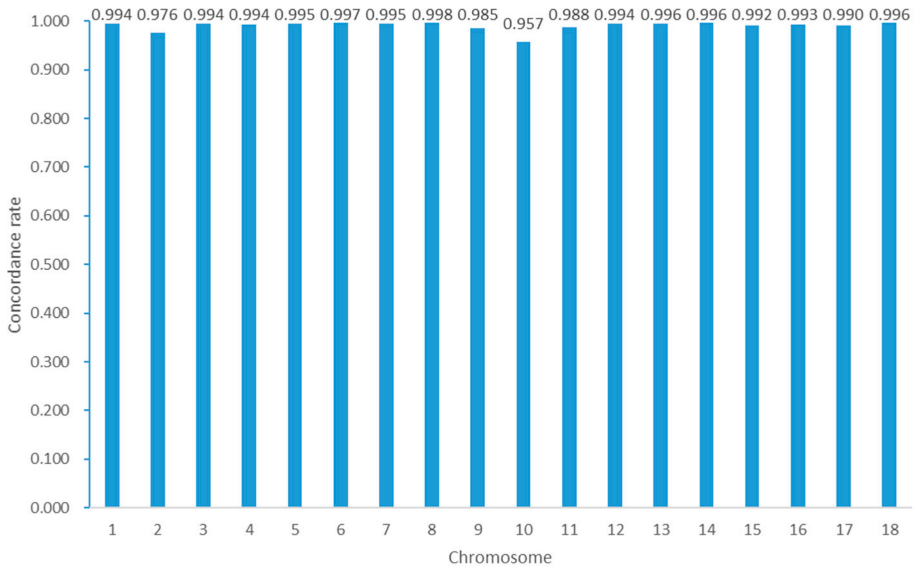

(b)

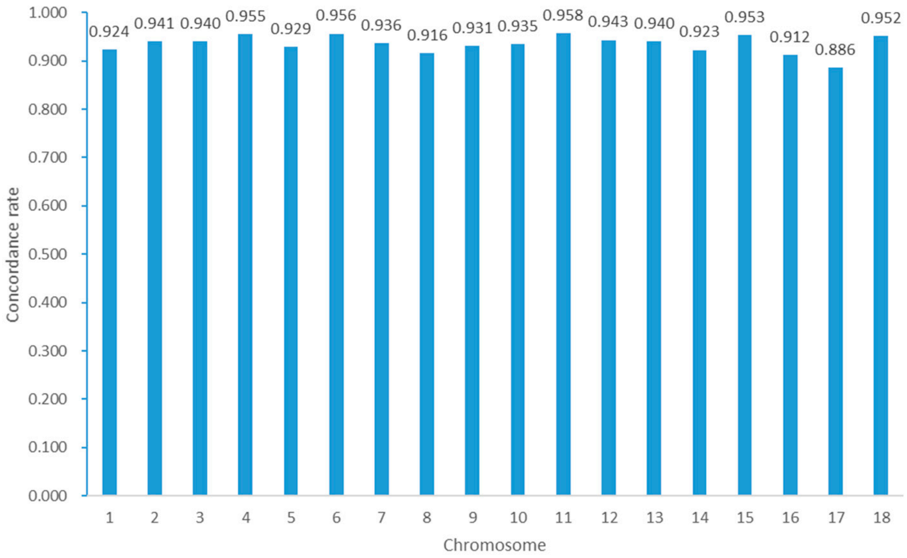


Figure S5. Allele concordance rate of each chromosome. (a) Allele concordance rate that imputing 50K Chip into 80K Chip; (b) Allele concordance rate that imputing 80K Chip into sequencing data.

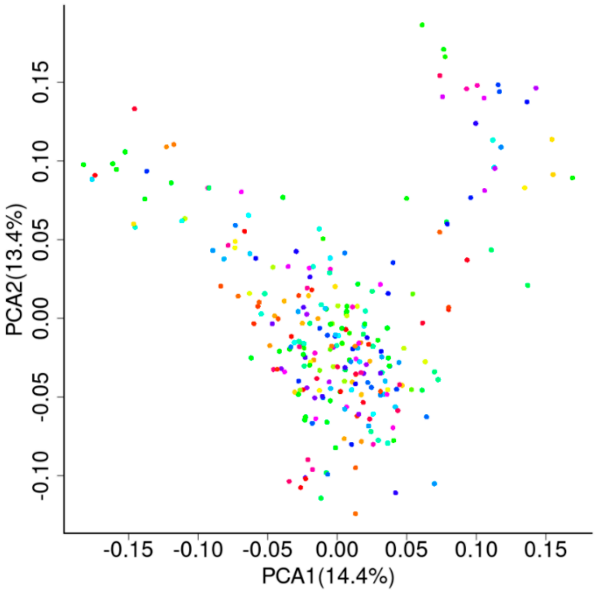


Figure S6. PCA plot of 270 Suhuai pigs by Chip data. Each point in the graph represents an individual.

(a)

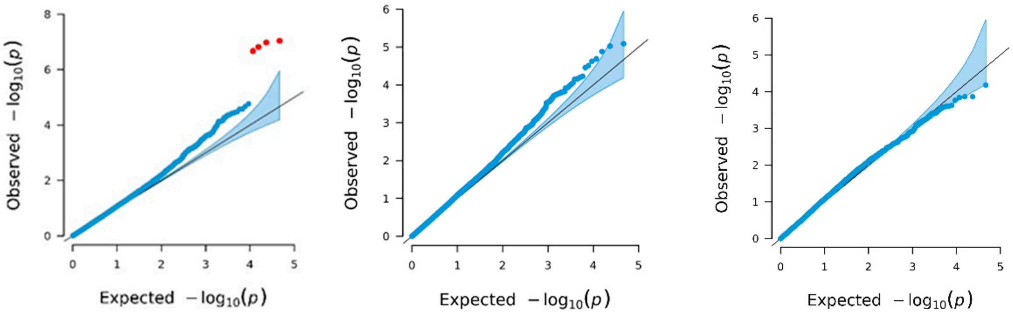


(b)

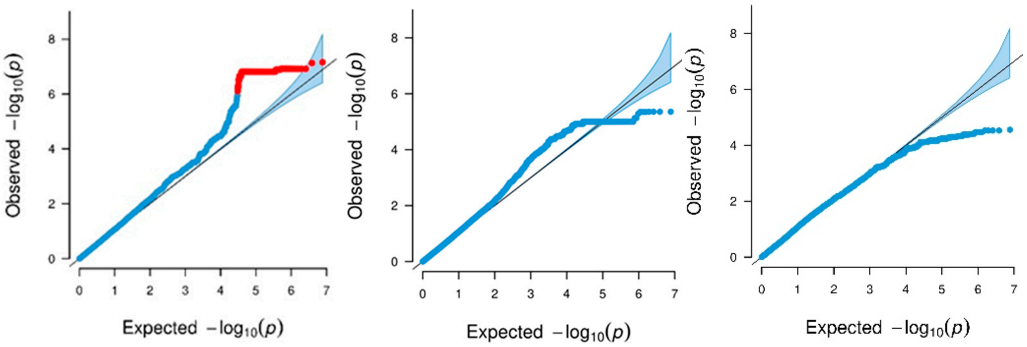


Figure S7. Q-Q plots. (a) The data was from Chip data. (b) The data was from imputed data. The plots from left to right were VL, VW and VA, respectively.
